# Estimating *R*_0_ from early exponential growth: parallels between 1918 influenza and 2020 SARS-CoV-2 pandemics

**DOI:** 10.1093/pnasnexus/pgac194

**Published:** 2022-09-17

**Authors:** Grant Foster, Bret D Elderd, Robert L Richards, Tad Dallas

**Affiliations:** Department of Biological Sciences, University of South Carolina, Columbia, SC 29208, USA; Department of Biological Sciences, Louisiana State University, Baton Rouge, LA 70803, USA; Department of Biological Sciences, Louisiana State University, Baton Rouge, LA 70803, USA; Department of Biological Sciences, Louisiana State University, Baton Rouge, LA 70803, USA; Department of Biological Sciences, University of South Carolina, Columbia, SC 29208, USA; Department of Biological Sciences, Louisiana State University, Baton Rouge, LA 70803, USA

**Keywords:** COVID-19, pandemic influenza, infectious diseases, public health

## Abstract

The large spatial scale, geographical overlap, and similarities in transmission mode between the 1918 H1N1 influenza and 2020 SARS-CoV-2 pandemics together provide a novel opportunity to investigate relationships between transmission of two different diseases in the same location. To this end, we use initial exponential growth rates in a Bayesian hierarchical framework to estimate the basic reproductive number, *R*_0_, of both disease outbreaks in a common set of 43 cities in the United States. By leveraging multiple epidemic time series across a large spatial area, we are able to better characterize the variation in *R*_0_ across the United States. Additionally, we provide one of the first city-level comparisons of *R*_0_ between these two pandemics and explore how demography and outbreak timing are related to *R*_0_. Despite similarities in transmission modes and a common set of locations, *R*_0_ estimates for COVID-19 were uncorrelated with estimates of pandemic influenza *R*_0_ in the same cities. Also, the relationships between *R*_0_ and key population or epidemic traits differed between diseases. For example, epidemics that started later tended to be less severe for COVID-19, while influenza epidemics exhibited an opposite pattern. Our results suggest that despite similarities between diseases, epidemics starting in the same location may differ markedly in their initial progression.

Significance Statement We often assume knowledge about historical epidemics can help establish reasonable expectations regarding the magnitude and spread of outbreaks of novel diseases with similar characteristics. However, the rarity of novel epidemics that span large spatial scales has made this assumption difficult to evaluate empirically, especially in the context of a global pandemic. Our study tests this assumption by comparing the initial outbreak dynamics of the 1918 H1N1 influenza and 2020 SARS-CoV-2 pandemics occurring in the same set of United States cities by quantifying R0, the reproductive rate of disease spread, for each city. Our results highlight how epidemic progression in a given location may differ markedly across diseases despite similarities in transmission mode.

## Introduction

No two epidemics are the same; stochastic effects can cause two epidemic trajectories to diverge even under otherwise identical circumstances, and variation in both pathogen traits and population-level factors can further affect epidemic outcomes. Understanding sources of variability and how they may affect key disease parameters is of paramount importance early in an outbreak. Chief among these key disease parameters is the basic reproduction number (*R*_0_), corresponding to the expected number of secondary cases generated by a single infected individual in a wholly susceptible population (1). While the limited spatial scope of many novel disease outbreaks make characterization of the full *R*_0_ distribution of a given disease difficult, the spatial scale exhibited by the 1918 influenza and 2020 COVID-19 pandemics make them ideal systems in which to investigate how variation between epidemics affect the overall *R*_0_ distribution. Additionally, their geographical overlap and similarities in transmission mode allow us to compare epidemic progression of each disease in the same city, providing insight into the influence of population-level factors on the spread of each disease similarly.

*R*_0_ itself is not an innate biological character of a pathogen, but rather conveys information about how a disease is transmitted through a given population. Consequently, *R*_0_ is affected by the traits of both the pathogen and host population (2). While pathogen traits may control the range of possible *R*_0_ values, the observed *R*_0_ may also be influenced by population characteristics (3,4). Variation in characteristics such as densities, contact structure, and mobility may all affect the probability of coming into contact with infected individuals and lead to interpopulation variability in *R*_0_. While understanding this spatiotemporal variability may better inform control strategies (3), both the spatial and temporal scope of any novel disease outbreaks are often limited. As the largest two respiratory pandemics of the 20th and 21st centuries, epidemics of H1N1 influenza and COVID-19 provide an opportunity both to better characterize the variability of *R*_0_ for each disease as well as investigate population characteristics correlated with an individual epidemic’s specific *R*_0_. Despite being caused by different viral families, the similarity of 1918 pandemic influenza and COVID-19 in transmission mode, global scope, and nonpharmaceutical interventions (NPIs) used to combat both pandemics have already inspired a number of comparative studies (5–7).

In order to better understand factors that may be correlated with variation in infection dynamics, we estimate initial *R*_0_ for both 1918 Pandemic influenza and COVID-19 using mortality data in 43 cities in the United States under a Bayesian hierarchical framework. By analyzing the first three weeks of mortality of multiple outbreaks of each disease across the same set of cities, we are able to both provide a general characterization of each disease’s associated *R*_0_ distributions and make novel comparisons across diseases. Despite the passing of a century, cities may retain historical similarities (e.g. patterns of movement, household size distribution), which relate to pathogen transmission factors, suggesting that transmission patterns from one epidemic may then be predictive for future epidemics with similar transmission modes. Due to their global scopes, together the 1918 influenza and COVID-19 pandemics form a dyadic system ideal for evaluating this assumption. We find that—despite occurring in the same locations—there is a surprising lack of concordance both between *R*_0_ estimates for the 1918 influenza and COVID-19 outbreaks. The diseases also exhibit opposing patterns in the effects of both population size and epidemic timing on *R*_0_.

## Results

### *R*_0_ distributions

**Fig. 1.**
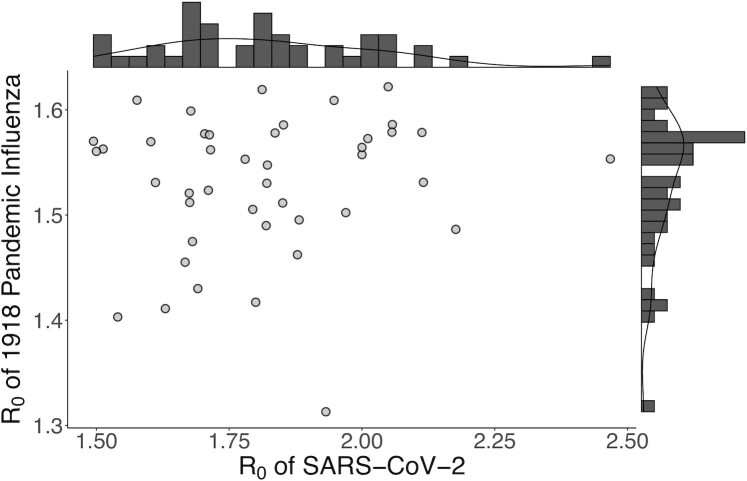
*R*_0_ estimates for 43 US cities for both pandemics based on early exponential growth rate. Despite occurring in the same location, median *R*_0_ estimates were not significantly correlated between pandemics (*r* = 0.114, *P*-value = 0.465).

In COVID-19 models only incorporating city identity, estimates of *R*_0_ ranged from 1.49 (Spokane, WA, USA) to 2.46 (New York, NY, USA), with a median value of 1.82 Fig. 1. For influenza, estimates of *R*_0_ ranged from 1.25 (Atlanta, GA, USA) to 1.60 (New Orleans, LA, USA), with a median value of 1.54. While most estimates of *R*_0_ for COVID-19 clustered about the median, the distribution of *R*_0_ for influenza was slightly bimodal. A likelihood ratio test comparing whether the *R*_0_ distribution was unimodal or bimodal confirmed that, indeed, *R*_0_ for influenza was bimodal (*P* < 0.05); maximum likelihood estimation found lower and upper modes of 1.48 and 1.55. In contrast, COVID-19 exhibited an unimodal *R*_0_ distribution (*P* > 0.05).

### Comparing across pandemics

Despite the similarity in transmission mode between the diseases, median estimates of *R*_0_ for the 1918 influenza and COVID-19 are uncorrelated across cities (*r* = 0.114, *P*-value = 0.465) Fig. 1. *R*_0_ estimates for COVID-19 epidemics were positively related to city size; models incorporating the effect of population size on *R*_0_ converged on 80% credible ranging from 0.130 to 0.242 per log-transformed number of people. In contrast, population size had no relationship with *R*_0_ for influenza; 80% credible intervals for its effect on *R*_0_ included 0, ranging from −0.049 to 0.059. Log change in population size between outbreaks was also uncorrelated with both the raw and standardized differences in median *R*_0_ estimates between the two diseases (*P*-value = 0.577 and 0.555, respectively).

### The effect of outbreak timing

**Fig. 2.**
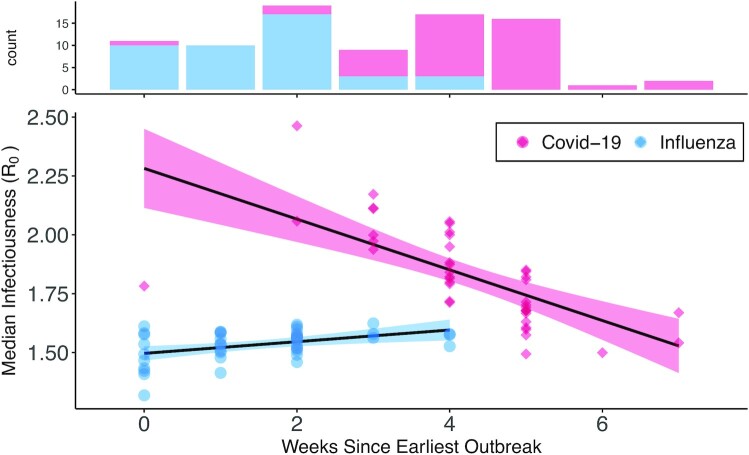
Relationship between epidemic timing across cities and median *R*_0_ estimate. For COVID-19 (pink), epidemics that occur later tend to be less severe; 80% credible intervals of the effect of epidemic start date range from −0.157 to −0.099. We see the opposite pattern for pandemic influenza (blue), for which the most severe epidemics tend to occur later in the year; 80% credible intervals of the effect of epidemic start date range from 0.049 to 0.121. Shaded polygons represent 95% CI of linear model.

Estimates of *R*_0_ for a given city were related to timing of outbreak emergence for both diseases, but the relationships differed in sign and strength Fig. 2. For COVID-19, epidemics starting later in the year tended to progress slower and have a lower *R*_0_. Models incorporating the fixed effect of outbreak timing converged on 80% credible intervals corresponding to weekly change in *R*_0_ of −0.157 to −0.099. In contrast, influenza exhibited a positive relationship with epidemic start date; coefficients for the fixed effect of outbreak timing converged on 80% credible intervals spanning 0.049 to 0.121, as new influenza epidemics tended to be more severe than their predecessors rather than less.

## Discussion

Our estimated distributions of *R*_0_ generally agree with studies utilizing other estimation methods, including those utilizing the same data sets (4,8). Contrary to expectations, city-specific estimates of *R*_0_ were not correlated across pandemics, nor were the differences in those estimates correlated with differences in population size. *R*_0_ estimates across pandemics were also correlated with site and epidemic-specific variables differently. *R*_0_ for COVID-19 was negatively correlated with outbreak timing (later outbreaks were less severe), while estimates for influenza were positively correlated with outbreak timing (later outbreaks grew faster). Population size was also positively correlated with *R*_0_ for COVID-19, while the two were uncorrelated for influenza. Median *R*_0_ estimates of influenza were bimodal, clustering around modes of 1.48 and 1.55. This bimodality may be due to differences in city-level epidemic control strategies; Hatchett, Mecher, and Lipsitch (2007) found that cities which implemented four or more NPIs early in their associated epidemic had less than half the peak weekly death rate than cities which responded later or with less intervention methods (9). Our higher cluster of *R*_0_ estimates may be comprised of cities who failed to intervene during the initial stages of epidemic progression, while the lower cluster may represent cities that took a more aggressive approach to minimizing spread, though we see no relationship between *R*_0_ mode clustering and the aforementioned NPI threshold (Supplementary Material).

Despite similarities in infection pathology, there were no associations between *R*_0_ estimates for influenza and COVID-19 for a given city. This may be due to the fact that key city characteristics change through the century as cities develop, or that traits may influence transmission of these viruses in weak or different ways despite their similarities. Through the past century many American cities have changed in different ways and at different rates. As the overall US population has increased, the population size rank order of some cities has remained relatively constant (e.g. New York, NY, USA), while others have drastically increased (e.g. Nashville, TN, USA) or decreased (e.g. St. Louis, MO, USA). If transmission rate is dependent on population size as many compartmental models assume (1), then these shifts in relative population size may drive some of the divergence of *R*_0_ between outbreaks. Additionally, changing human behaviors may contribute to infection pattern differences. While we found no evidence for the effect of population size on pandemic influenza transmission, factors such as household composition, intercity travel patterns, frequency and scale of community gatherings, and popularity of public transportation could all affect the probability of an infectious contact. With ongoing infrastructure, demographic, and cultural changes in cities, these differences may ultimately impact epidemic progression as well, causing similar diseases to exhibited markedly different progression.

Alternatively, even if these relationships are conserved, the progression of pandemic influenza and COVID-19 may simply be influenced by different factors. While their geographic extents and transmission modes make comparison of the two diseases natural, they differ markedly in pathology, morbidity, and infectivity, and as such may be affected by population-level traits differently. For example, *R*_0_ scales positively with population size for COVID-19 while the two are not associated for influenza. This could possibly be due to differences in types of contacts (e.g. duration, proximity) required for each virus to transmit. If the frequency of certain types of contact scale more or less strongly with density then we may expect a similarly stronger or weaker effect of population size for a given virus. Future work focusing on how disease and population traits interact to influence disease progression may ultimately allow us to develop predictions and response strategies better tailored to individual locations.

Estimated *R*_0_ for COVID-19 was negatively related to epidemic start date, while influenza exhibited the opposite relationship. The former pattern may be due to the adoption of human responses to disease outpacing spatial disease spread. For example, adoption of NPI strategies such as mask-usage and social distancing spread throughout the United States in response to early COVID-19 outbreaks may have reduced early transmission in later epidemics. Alternatively, this reduction could be more to do with mortality than transmission; a decrease in mortality rate as physicians gain and share experience in treating the disease could manifest as a reduction in *R*_0_ over the course of a pandemic. Improvements in disease surveillance may have also lead to earlier diagnosis and improved survival rates as the epidemic progressed. This pattern may instead be explained through changes in viral-traits rather than population ones, perhaps through evolutionary differences in epidemic strains. The fact that COVID-19 exhibited a negative relationship between *R*_0_ and epidemic timing while influenza did not may be due to the rate at which the aforementioned population responses were adopted. Advancements in information technology and pandemic preparedness ideally let us respond to potential epidemics better and faster, allowing us to slow early spread.

In contrast, the positive relationship between influenza *R*_0_ and epidemic timing may be due to larger-scale seasonal changes. Similar to nonpandemic strains of influenza, transmission of pandemic influenza may be improved by seasonal environmental factors affecting viral persistence such as lower temperatures, relative humidity, and UV exposure (10). This increase in viral persistence could correspond to higher infectiousness, resulting in more rapid spread in epidemics occurring later in the year. Alternatively the positive change may be more behavioral; as people begin to spend more time indoors out of the cold weather, there may be increased potential for infectious contacts.

As the two largest respiratory pandemics to occur since the start of the 20th century, making comparisons between the pandemic influenza and COVID-19 is a point of interest. By leveraging multiple epidemic time series across a wide geographic extent we characterize the distribution of *R*_0_ of these pandemics in the United States, providing insight into how variability in population characteristics relates to variability in disease transmission patterns. Despite similar transmission modes, these diseases exhibit contrasting relationships with epidemic timing and population size. We intend for these correlative patterns to inspire future work aimed at elucidating mechanisms by which city-level traits may influence epidemic dynamic. Further understanding of these mechanisms may improve our ability to predict and understand epidemic variability across spatiotemporal scales.

## Materials and methods

Estimates of the exponential rate of increase in infections (*r*) were calculated using mortality data from the first three weeks since epidemic onset in 43 US cities using Bayesian hierarchical models, where epidemic onset is defined as the first day with 10 confirmed COVID-19 deaths for SARS-CoV-2 and the start of the first three consecutive weeks in late 1918 with positive mortality above baseline for pandemic influenza. These time periods constituted the initial COVID-19 wave and the second, largest pandemic influenza wave in the United States, respectively. These estimates were then converted to *R*_0_ (11, 12) assuming infectious period estimates of 5.4 and 2.83 days for SARS-CoV-2 (13) and influenza (14), respectively. All models included city identity and a fixed intercept; when included, population size and epidemic start date were incorporated as fixed effects. Model fitting was performed using Markov Chain Monte-Carlo (MCMC) methods as implemented in JAGS (15) without chain thinning (16). For detailed methods, see Supplementary Material.

## Supplementary Material

pgac194_Supplemental_Files

## Data Availability

Our analysis uses publicly available mortality data for COVID-19 (17) and pandemic influenza (18). All code and associated data necessary to recreate this analysis are publicly available on figshare at https://doi.org/10.6084/m9.figshare.19763074.v2.
