# Supplementary material for: Estimating R0 from early exponential growth: parallels between 1918 influenza and 2020 SARS-CoV-2 pandemics
Source: PNAS Nexus. 2022 Sep 17;1(4):pgac194. doi: 10.1093/pnasnexus/pgac194 (PMC9802102; doi:10.1093/pnasnexus/pgac194)
Supplement: pgac194_Supplemental_Files [file pgac194_supplemental_files.zip › PNASNEXUS-PNASNEXUS-2022-00583-T-s01.pdf]

Supporting Information

September 12, 2022

Supplemental Methods

Data and Code Accessibility

All code and associated data necessary to recreate this analysis are publicly available on figshare: https://doi.org/10.6084/m9.figshare.19763074.v2

Estimation of r and R_0

For the basic SIR model, $\frac{dI}{dt} \approx (\beta - \gamma)I$, when linearized about the disease-free equilibrium [1], approximates exponential growth. During this exponential growth phase, the exponential rate of increase, r, is equal to $\beta - \gamma$, and infectious individuals at time t can be estimated according to the equation $N(t) = N_0 e^{rt}$. Solving for r, $r = ln(N_0)/t$ allows us to estimate r using linear regression. As described in Wallinga and Lipsitch (2007), when derived from the Lotka-Euler equation $R = \int_0^\infty n(a)\, da$, where $n(a)$ is the rate of offspring production (secondary infections in the disease case) of a infected of age a. This rate can then be normalized to a generation interval distribution characterized by a unique moment generating function, such that $R = \frac{1}{M(-r)}$. Assuming an exponentially distributed generation time with a mean T_c, $R = 1 + \frac{r}{\gamma}$, where $\gamma = 1/T_c$[2], and as such Assuming infections are equally probable throughout the infectious period (γ^{-1}), then we assume $E(\gamma^{-1})$ = Mean Serial Interval (SI), and in concert with our previous assumption of an exponentially distributed generation time our equation for R_0 becomes $R_0 = r \times SI + 1$. Estimates of r were transformed into R_0 assuming serial intervals of 2.83 and 5.4 days for flu[3]and SARS-CoV-2[4], respectively.

By virtue of the fact that $R_0 = \frac{\beta}{\gamma}$ for the SIR model, we can convert estimates of r to R_0 using the equation $R_0 = r/\gamma + 1$ [1]. Further assuming an exponentially distributed infectious period, then $E(\gamma^- 1) = 2\times$ Mean Serial Interval (SI), and as such our equation for R_0 becomes $R_0 = 2r \times SI + 1$. Estimates of r were transformed into R_0 assuming serial intervals of 2.83 and 5.4 days for flu[3] and SARS-CoV-2 [4], respectively.

We estimate r from disease mortality data, assuming deaths $\propto I$ at a fixed proportion throughout each epidemic. While variations in SARS-CoV-2 strain virulence and prevalence likely invalidate this assumption over the course of the full pandemic, this assumption is reasonable given our focus on earliest progression of epidemic outbreaks, prior to the emergence of variants of concern in the US. After log transforming death time series for each epidemic we perform both Bayesian fixed and random effects linear regression, holding the intercept constant but allowing the slope to vary across outbreaks. Markov Chain Monte-Carlo chains were run using JAGS[5]; each regression was run using 4 chains ran for 10,000 time steps. The first 1,000 time steps were discarded for burn-in; chains were analyzed without thinning[6]. All models included the fixed effect of city identity on slope and a random intercept; best fit models did not include an interaction term between slope and intercept. Additional models incorporated the effect population data and relative outbreak start date on the regression slope; 80% credible intervals for these parameters were constructed via quantiles of converged MCMC samples.

Data

Weekly death data for 1918 Pandemic influenza outbreaks for 50 United States cities were obtained from Collins et al.[7] influenza deaths were recorded as deaths above background mortality as defined as median deaths from 1910-1916. For each city, the first three consecutive weeks with positive mortality above baseline were used to calculate epidemic slope in our exponential growth model. County-level

daily death data was obtained for COVID-19 from The New York Times[8]. For each city in our study, we summed both the total covid deaths and population from all counties that at least partially contain a given city limits. While this may include a significant metropolitan population outside city limits in cities such as Atlanta or Los Angeles, we believe this method to be the best way to compare across data sets available to us. We defined the start of the epidemic as the first day confirmed Covid deaths exceeded 10. We then summed daily deaths into weekly totals in order to directly compare to influenza, for which only weekly records were available, and then used the same criteria of the first three consecutive weeks to calculate epidemic slope.

For Covid-19, this study period represents the first epidemic wave in the United States following the introduction of SARS-Cov-2 in early 2020. While viral strain diversity may have contributed to some variation in infection patterns, our study period likely predates the earliest spread variant of concern (Alpha, or B.1.1.7) in the US by a number of months[9]. As such, we believe our analysis is unlikely be greatly affected by strain variation in the initial Covid-19 outbreaks. For influenza, our study period captures the second wave, with the first occurring earlier in the spring at 1918. After a relatively mild and low morbidity "herald wave," occurring in Spring of 1918, the majority of US infections and deaths occurred during this Fall second wave. While some cross-immunity from the initial spring wave may have potentially have biased our R_0 estimates for influenza[10] downward, given the limited size of the Spring outbreak in the United States we believe our analysis to still be broadly applicable.

City population size was retrieved from the 1910 United States census[11] for influenza. County population estimates for 2019 were retrieved from the US Census Bureau Population Estimates API[12], accessed in R via the 'tidycensus' package[13].

Contribution of NPI on Influenza R_0 Modality

Analysis

In main text, we describe a pattern of bimodality in median R_0 estimates for influenza, with estimates clustering about upper and lower modes of 2.04 and 2.14, respectively. To investigate whether this modality may in part be driven by differences in city-level Non-Pharmaceutical Intervention (NPI) strategies, we compare our results to that of Hatchett, Mecher, and Lipsitch (2007), who investigated the effects of various NPI strategies on epidemic intensity of 1918 pandemic influenza in set of 17 American cities. We designated the 16 cities shared between our studies as either "fast responding" or "slow responding" based on whether they implemented 4 or more NPI measures before achieving a daily case fatality rate of 20 excess weekly deaths/100,000[14]. We found no relationship between NPI response speed and whether a given city was assigned to the upper or lower mode ($\chi^2 = 0.291, p = 0.590$). Logistic regression of the effect of posterior probability of assignment to a given mode was also unrelated to NPI response speed ($p = 0.940$).

References

[1] Junling Ma. Estimating epidemic exponential growth rate and basic reproduction number. *Infectious Disease Modelling*, 5:129–141, 2020.

[2] Jacco Wallinga and Marc Lipsitch. How generation intervals shape the relationship between growth rates and reproductive numbers. *Proceedings of the Royal Society B: Biological Sciences*, 274(1609):599–604, 2007.

[3] Laura Forsberg White and Marcello Pagano. Transmissibility of the influenza virus in the 1918 pandemic. *PLoS One*, 3(1):e1498, 2008.

[4] Balram Rai, Anandi Shukla, and Laxmi Kant Dwivedi. Estimates of serial interval for covid-19: A systematic review and meta-analysis. *Clinical epidemiology and global health*, 2020.

[5] Sarah Depaoli, James P Clifton, and Patrice R Cobb. Just another gibbs sampler (jags) flexible software for mcmc implementation. *Journal of Educational and Behavioral Statistics*, 41(6):628–649, 2016.

[6] William A Link and Mitchell J Eaton. On thinning of chains in mcmc. *Methods in ecology and evolution*, 3(1):112–115, 2012.

[7] Selwyn D Collins, Wade Hampton Frost, Mary Gover, and Edgar Sydenstricker. Mortality from influenza and pneumonia in 50 large cities of the united states, 1910-1929. *Public Health Reports*, 45:2277–2328, 1930.

[8] New York Times. Coronavirus (covid-19) data in the united states. https://github.com/nytimes/covid-19-data, Retrieved November, 2020.

[9] Prabasaj Paul, Anne Marie France, Yutaka Aoki, Dhwani Batra, Matthew Biggerstaff, Vivien Dugan, Summer Galloway, Aron J Hall, Michael A Johansson, Rebecca J Kondor, et al. Genomic surveillance for sars-cov-2 variants circulating in the united states, december 2020–may 2021. *Morbidity and Mortality Weekly Report*, 70(23):846, 2021.

[10] John M Barry, Cécile Viboud, and Lone Simonsen. Cross-protection between successive waves of the 1918–1919 influenza pandemic: epidemiological evidence from us army camps and from britain. *The Journal of infectious diseases*, 198(10):1427–1434, 2008.

[11] Campbell Gibson. *Population of the 100 Largest Cities and Other Urban Places in the United States:*. Population Division, US Bureau of the Census, 1998.

[12] U.S. Census Bureau. Population estimates api: Vintage 2019, 2021.

[13] Kyle Walker and Matt Herman. *tidycensus: Load US Census Boundary and Attribute Data as 'tidyverse' and 'sf'-Ready Data Frames*, 2022. R package version 1.2.1.9000.

[14] Richard J Hatchett, Carter E Mecher, and Marc Lipsitch. Public health interventions and epidemic intensity during the 1918 influenza pandemic. *Proceedings of the National Academy of Sciences*, 104(18):7582–7587, 2007.
